# Löfgren Syndrome: A Case Report and Treatment Challenge

**DOI:** 10.7759/cureus.59597

**Published:** 2024-05-03

**Authors:** João Miguel Peixoto, Diogo Leal, Dilva Silva, Lèlita Santos

**Affiliations:** 1 Internal Medicine, Centro Hospitalar e Universitário de Coimbra, Coimbra, PRT

**Keywords:** steroid therapy, diagnosis, löfgren syndrome, erythema nodosum, sarcoidosis

## Abstract

Sarcoidosis is an inflammatory and immune-mediated multisystemic disorder of unknown etiology, characterized by the presence of non-caseating granulomas, impacting various organs. This indolent condition manifests with numerous nonspecific symptoms and lacks a definitive diagnostic test, typically requiring histopathologic confirmation. However, a distinct and more readily diagnosable form of sarcoidosis does exist. The Löfgren syndrome (LöS) is characterized by the triad of erythema nodosum (EN), bilateral hilar lymphadenopathy, and symmetrical inflammatory arthralgias or arthritis. The simultaneous presence of these elements obviates the necessity for a biopsy. Predominantly affecting women in their second and third decades of life, this syndrome generally carries a favorable prognosis with spontaneous resolution or the requirement for a nonsteroidal anti-inflammatory drug (NSAID) alone. Despite its rarity, in particular cases, the treatment can be more challenging. This article presents a case study of LöS in a young woman, whose more aggressive disease course led to the need for steroidal therapy.

## Introduction

Sarcoidosis is a multisystemic inflammatory/immune-mediated disease characterized by the presence of non-caseating granulomas and the potential to impact virtually every organ [1,2]. While it can affect individuals of all races, there is notable variability in annual incidence and prevalence among ethnic groups, with a higher occurrence observed in African Americans and Scandinavians [1,3]. Sarcoidosis can also manifest at any age, but it exhibits a distinct peak incidence between 20 and 39 years, with a higher prevalence in females [2-4].

It is considered an idiopathic disorder, and extensive research suggests that it arises from an interplay between genetic susceptibility and environmental exposures (infections in particular). This interaction triggers an antigen presentation reaction by macrophages to T helper lymphocytes (mainly CD4), leading to an inflammatory and immune response, culminating in the formation of non-caseating granulomas - the distinctive hallmark of sarcoidosis, although not pathognomonic [1,4,5].

Clinically, sarcoidosis manifests as a condition involving various organs and tissues, thus displaying a broad and heterogeneous spectrum of clinical manifestations. This diversity makes its diagnosis more challenging, with the need for several differential diagnoses [1,2]. Notably, nearly half of the sarcoidosis patients initially present without symptoms, the diagnosis being frequently incidental following the discovery of hilar lymphadenopathies on chest imaging (X-ray or CT scan) [1,3,4]. Pulmonary involvement, termed pulmonary sarcoidosis, predominates with enlarged hilar and mediastinal lymph nodes constituting the primary abnormality observed in approximately 90% of cases. Subsequent parenchymal involvement ranges from pulmonary infiltrates to fibrosis, albeit less commonly encountered [1,2]. While the skin and eyes rank as the second and third most commonly affected organs, respectively, sarcoidosis can also affect the nervous system, heart, liver, and kidneys, occasionally presenting with more severe manifestations [1-3].

Cutaneous sarcoidosis may present with different patterns, usually displaying non-caseating granulomas, like lupus pernio, verrucous scaly spots, ichthyosis/dry scaly skin of legs, subcutaneous lumps or bumps, hypopigmented patches, or with nonspecific lesions, predominantly erythema nodosum (EN).

EN appears as painful, erythematous, slightly elevated skin nodules, primarily located on the anterior surface of the legs, which biopsy reveals as septal panniculitis. However, it is important to recognize that it can arise from various causes beyond sarcoidosis (infections, drug reactions, enteropathies, and malignancies, particularly lymphoma) [1,2,6,7].

When accompanied by bilateral hilar lymphadenopathy and symmetrical inflammatory arthralgias or arthritis, it usually represents an acute form of sarcoidosis known as Löfgren syndrome (LöS) [7,8]. LöS constitutes 5-10% of sarcoidosis cases, yet it is indeed a distinct phenotype of the disease [5,7]. Unlike sarcoidosis, which typically has a more gradual onset and often progresses to chronicity, LöS develops acutely and tends to be self-limited. It is more common in women, while men are more likely to present with arthritis only [3,5,9]. Constitutional symptoms, such as fever, malaise, and fatigue, may also be present [1,5].

## Case presentation

**Figure 1:**
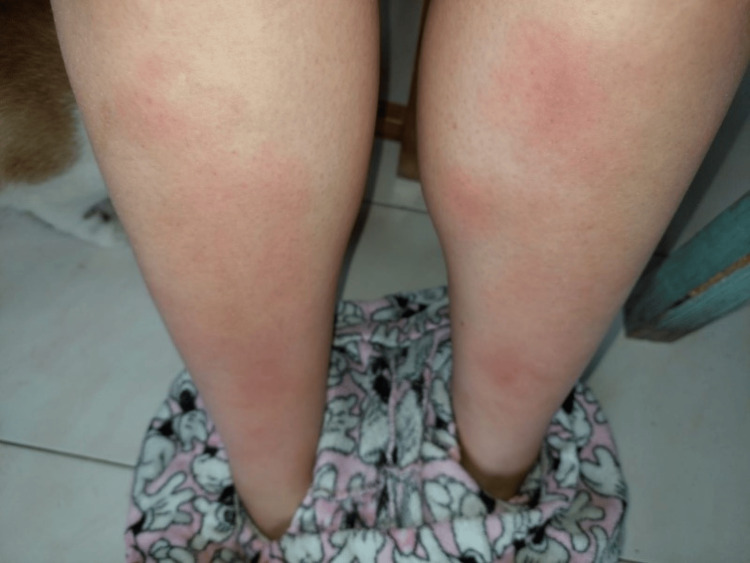
Patient’s skin lesions compatible with EN EN, erythema nodosum

**Figure 2:**
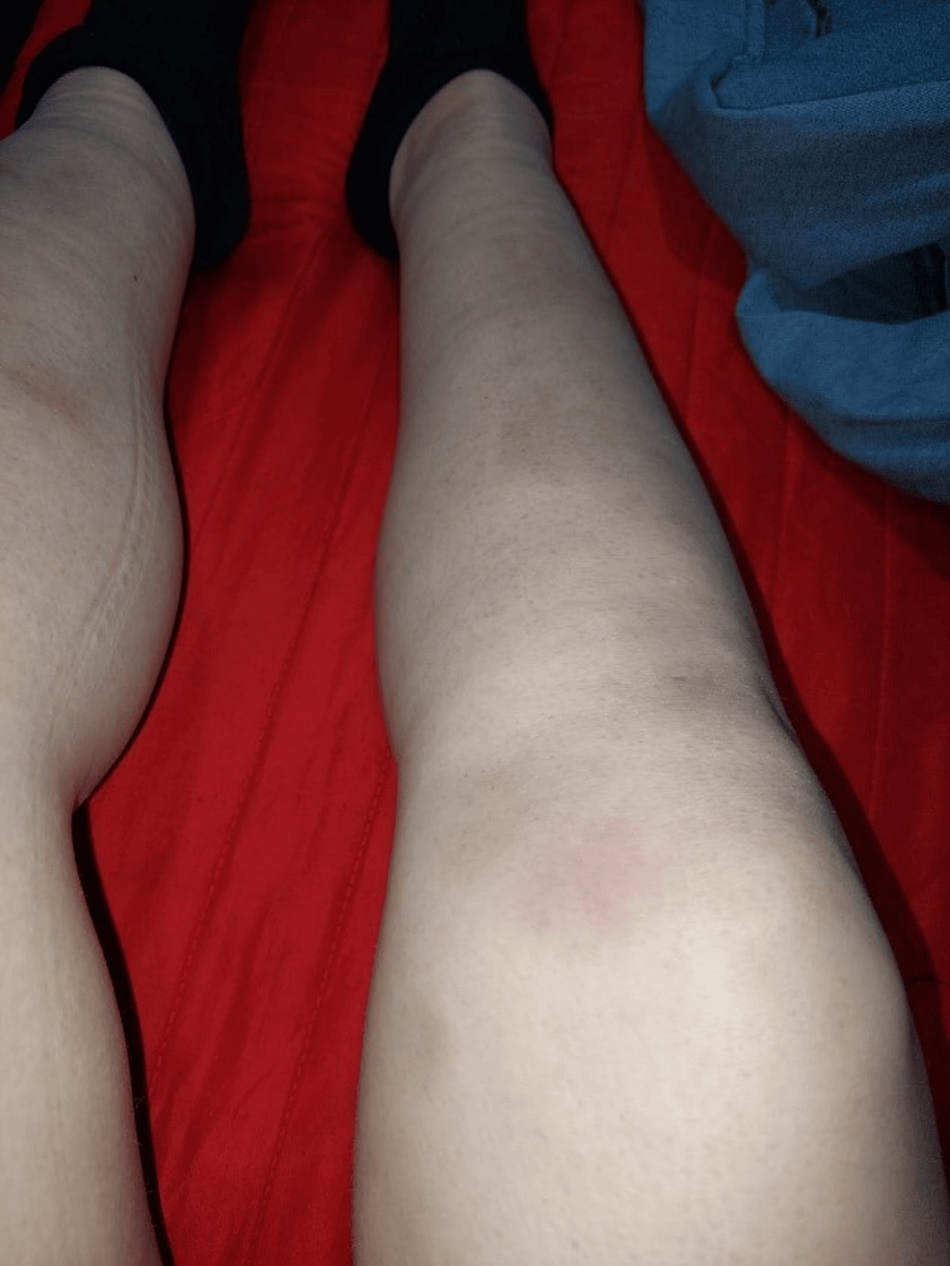
Nodules of EN and patient’s swollen ankles EN, erythema nodosum

A 27-year-old woman, with hypothyroidism due to Hashimoto's thyroiditis, under treatment with Levothyroxine, was referred for an Internal Medicine consultation due to the presence of tender, erythematous cutaneous lesions located on the anterior surface of both legs, indicative of EN (Figures 1, 2). Additionally, she was experiencing swelling and pain in both ankles, displaying an inflammatory pattern (Figure 2), suggesting the presence of arthritis, which emerged two weeks after the onset of EN. The patient did not report any other symptoms, including fever, dyspnea, dry cough, or chest pain. 

**Figure 3:**
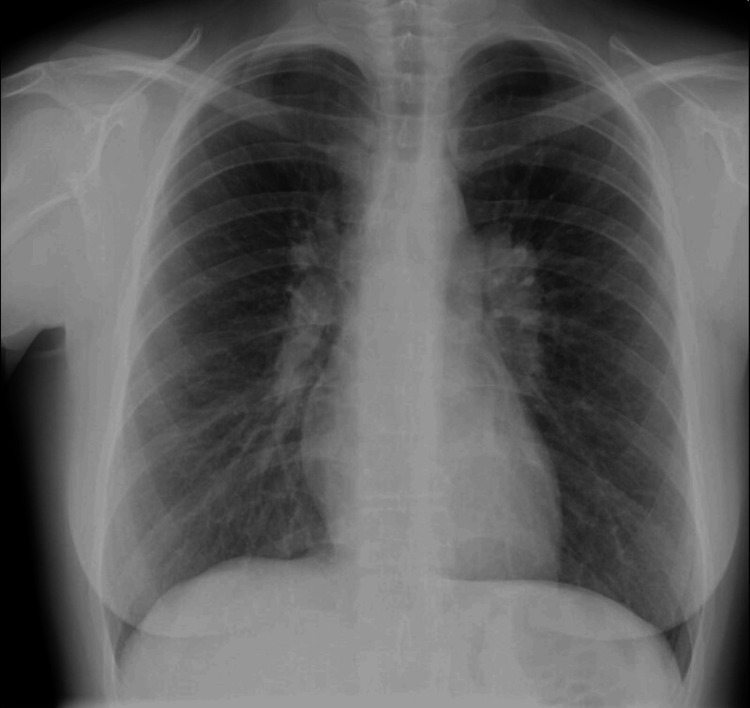
Thorax X-ray showing bilateral hilar lymphadenopathies

After an initial workup, which included laboratory tests and a thorax X-ray, the patient was treated with Naproxen 500 mg twice daily. Three weeks later, a significant worsening of arthralgia and a minimal improvement in EN were observed. Laboratory results showed a normal blood cell count and biochemistry panel, negative autoimmune profile, Interferon Gamma Release Assay (IGRA), serum protein electrophoresis, and immunofixation, but an increased ESR (42 mm/h), CRP (3.4 mg/dL), and serum angiotensin-converting enzyme (ACE) (92 U/L). Thorax X-ray findings displayed bilateral enlarged hilar regions, suggesting lymphadenopathies, with preserved lung parenchyma (Figure 3). A CT scan later confirmed these findings, and a bronchoscopy and bronchoalveolar lavage (BAL) were also performed, revealing a CD4/CD8 ratio of 9.8, and no other abnormalities, including isolation of mycobacteria.

Considering the clinical presentation and the exclusion of major potential causes, such as tuberculosis (TB), lymphoma, and autoimmune disorders, a diagnosis of LöS was established. Despite three weeks of treatment with a nonsteroidal anti-inflammatory drug (NSAID) showing no improvement, 20 mg daily of Prednisolone was initiated and gradually tapered over four weeks, resulting in symptom resolution. However, the disorder recurred upon complete steroid cessation, with EN and arthritis of both ankles, which required a new cycle of Prednisolone treatment. A more gradual tapering regimen over a six-month period led to a full clinical recovery, with normalization of the ESR, CRP, and ACE. 

## Discussion

The diagnostic approach to LöS diverges from that of sarcoidosis. The latter is typically a diagnosis of exclusion and mandates histopathological confirmation of the non-caseating granulomas within at least one affected tissue. Therefore, two more conditions are essential to the diagnosis of sarcoidosis: a clinical presentation consistent with the disorder, and the exclusion of alternative etiologies, notably TB, other mycobacterial infections, fungal infections, lymphoma, other malignancies, systemic vasculitis (particularly granulomatosis with polyangiitis), and connective tissue diseases, predominantly lupus [1,3,6].

Conversely, LöS represents an exception, as it is typically diagnosed based on its characteristic clinical profile, with a specificity of 95%, thereby obviating the necessity for histopathological examination and other additional tests [5,6,8]. Nonetheless, certain diseases must still be systematically excluded (TB, lymphoma, reactive arthritis, enteropathies, systemic vasculitis, and lupus).

Certain tests may support the diagnosis of both sarcoidosis and LöS, such as elevated levels of serum ACE and/or the presence of hypercalcemia and hypercalciuria. However, given their low specificity and sensitivity, their absence or presence alone does not definitively exclude or confirm the disease [3,5]. CT scans to confirm hilar lymphadenopathies are also deemed unnecessary, although it may be requested to better assess lung parenchyma and rule out alternative diagnoses [3,4]. Similarly, bronchoscopy and BAL may be employed to exclude other differentials, particularly TB, with the presence of a CD4/CD8 ratio greater than 3.5 potentially suggestive of sarcoidosis [3].

In this case, despite the highly indicative clinical presentation, we decided to perform a CT scan and BAL, alongside additional blood tests, revealing abnormalities supportive of the hypothesis of sarcoidosis/LöS.

In contrast to sarcoidosis, LöS typically manifests as a self-limited disease with a favorable prognosis, characterized by spontaneous resolution of symptoms within several months to a year, in approximately 90% of patients. EN typically resolves within six to eight weeks [5,8,9].

Treatment primarily focuses on symptom relief, often achieved through the use of NSAIDs. In more severe cases (approximately 10% of patients), Prednisolone (20 mg daily or less) may be necessary to induce remission. However, tapering can be expedited within one to two months as symptoms often resolve rapidly [5,8,9]. Recurrence is exceedingly rare [5,9]. In this case, despite the initial improvement, the patient not only needed steroid therapy to achieve remission, but also showed signs of recurrence, requiring a slower steroid tapering. 

## Conclusions

LöS syndrome is an acute form of sarcoidosis. The presence of the typical triad (EN, bilateral hilar lymphadenopathy, and symmetrical inflammatory arthralgias or arthritis) is usually enough to make the diagnosis, excluding the need for histopathological confirmation. Treatment often targets pain control, without the need for steroid therapy. This case report holds significance as it highlights a challenging case of LöS, underscoring the potential need for treatment adaptations in cases of refractoriness and recurrence.
